# Supplementary material for: Clustering-based spatial analysis (CluSA) framework through graph neural network for chronic kidney disease prediction using histopathology images
Source: Sci Rep. 2023 Aug 5;13:12701. doi: 10.1038/s41598-023-39591-8 (PMC10404289; doi:10.1038/s41598-023-39591-8)
Supplement: Supplementary file 1 — Supplementary Information. [file 41598_2023_39591_MOESM1_ESM.pdf]

SUPPLIMENTARY MATERIAL

Table S1. Clinical data for 57 CKD patients[1].

Patients	Age	Gender	Race	Diagnosis	eGFR	UPC
1	33	Female	White/Caucasian	Lupus Class III	96.87	2.27
2	39	Female	White/Caucasian	Lupus Class V, MPGN	90.93	1.46
3	34	Female	White/Caucasian	Lupus Class V	118.92	1.9
4	32	Female	Black/African American	Lupus Class III, Lupus Class IV	113.06	0.43
5	24	Female	Asian/Asian American	Lupus Class III	121.27	1.87
6	49	Male	White/Caucasian	Minimal Change	70.58	7.71
7	23	Female	White/Caucasian	HSP	122.12	0.48
8	46	Male	White/Caucasian	FSGS, IgA Nephropathy	47.31	2.2
9	19	Female	American Indian/Alaskan Native	FSGS	119.68	0.9
10	66	Female	White/Caucasian	Diabetic nephropathy	26.62	4.6
11	68	Female	White/Caucasian	Minimal change, FSGS	27.63	3.36
12	20	Male	Black/African American	FSGS	70.82	1.27
13	5	Male	White/Caucasian	FSGS	118.9	1.86
14	41	Female	Black/African American	Lupus Class V, hypertensive nephropathy	43.59	0.15
15	51	Female	Black/African American	Lupus Class III, lupus Class V	98.93	8.25
16	40	Female	White/Caucasian	Lupus nephritis	79.98	5.37
17	11	Female	White/Caucasian	HSP	160.93	1.72
18	38	Female	White/Caucasian	IgA Nephropathy	93.52	5.97
19	22	Female	White/Caucasian	Lupus Class II, MPGN	104.65	2.69
20	37	Female	Black/African American	Lupus Class V	83.35	2.48
21	48	Female	White/Caucasian	Lupus Class III	107.78	2.9
22	58	Female	White/Caucasian	FSGS	32.67	0.21
23	55	Male	Asian/Asian American	IgA Nephropathy, Hypertensive nephropathy	34.4	2.95
24	91	Male	White/Caucasian	Glomerular disease	22.96	2.23
25	45	Female	White/Caucasian	Membranous Nephropathy	105.13	3.9
26	56	Male	White/Caucasian	Glomerular disease	96.94	0.08
27	19	Female	Black/African American	Lupus class IV	95.905	1.65
28	80	Male	White/Caucasian	FSGS, tubulointerstitial disease	30.8	8.13
29	26	Female	Multiracial	Lupus class III, Lupus class IV	142.74	0.73
30	6	Male	White/Caucasian	IgA Nephropathy	131.41	3.41
31	26	Female	Black/African American	Lupus Class V	117.93	0
32	20	Female	White/Caucasian	Lupus Class II	109.43	0.06
33	51	Female	White/Caucasian	Lupus Class III	85.36	2.69
34	29	Female	White/Caucasian	Lupus Class V	109.48	2.19
35	45	Female	#N/A	Lupus Class V	111.95	1.56
36	30	Female	Black/African American	Lupus Class V	73.94	4.51
37	73	Male	White/Caucasian	Diabetic nephropathy, hypertensive nephropathy	66.24	1.49
38	59	Male				
39	37	Female	White/Caucasian	Diabetic nephropathy, hypertensive nephropathy, Tubulointerstitial disease	19.52	9.11
40	53	Female	White/Caucasian	Diabetic nephropathy	15.75	1.39
41	58	Male	White/Caucasian	Diabetic nephropathy	56.05	0.91
42	42	Female	White/Caucasian	Diabetic nephropathy	44.68	6.74
43	69	Female	Black/African American	Diabetic nephropathy	64.23	1.01
44	62	Male	White/Caucasian	Diabetic nephropathy	18.29	6.49
45	21	Female	White/Caucasian	Lupus Class V	163.67	3.48
46	29	Female	White/Caucasian	Lupus Class IV	123.17	0.99
47	7	Female	Asian/Asian American	Membranous Nephropathy	180.4	3.19
48	62	Male	White/Caucasian	IgA Nephropathy	53.47	0.31
49	71	Male	White/Caucasian	FSGS	46.17	2.74
50	36	Female	#N/A	IgA Nephropathy	114.22	0.43
51	37	Female	Asian/Asian American	Glomerular disease	114	0.42
52	31	Female	White/Caucasian	FSGS, IgA Nephropathy	51.27	1.72
53	51	Female	Black/African American	Membranous Nephropathy	91.94	6.31
54	67	Male	Black/African American	Diabetic nephropathy, Tubulointerstitial disease	25.88	5.54
55	48	Female	White/Caucasian	Diabetic nephropathy, Lupus Class II	44.32	8.42
56	59	Female	White/Caucasian	Diabetic nephropathy	25.87	8.53
57	48	Male	White/Caucasian	Diabetic nephropathy, FSGS	48.72	0.53

Table S2. Confusion matrix and AUC for the eGFR at the biopsy for the top 7 features

		Prediction	
		0	1
Actual	0	34	2
	1	1	20
**Accuracy**		0.95	
**AUC**		0.96	
**95% CI**		0.90 – 1.0	

0: eGFR ≥ 60, **1:** eGFR < 60, **CI**: confidence interval

Table S3. Confusion matrix and AUC for the eGFR in one year for the top 7 features

		Prediction	
		0	1
Actual	0	25	5
	1	4	23
**Accuracy**		0.84	
**AUC**		0.85	
**95% CI**		0.76 – 0.95	

0: eGFR slope < 0, **1:** eGFR slope ≥ 0, **CI**: confidence interval

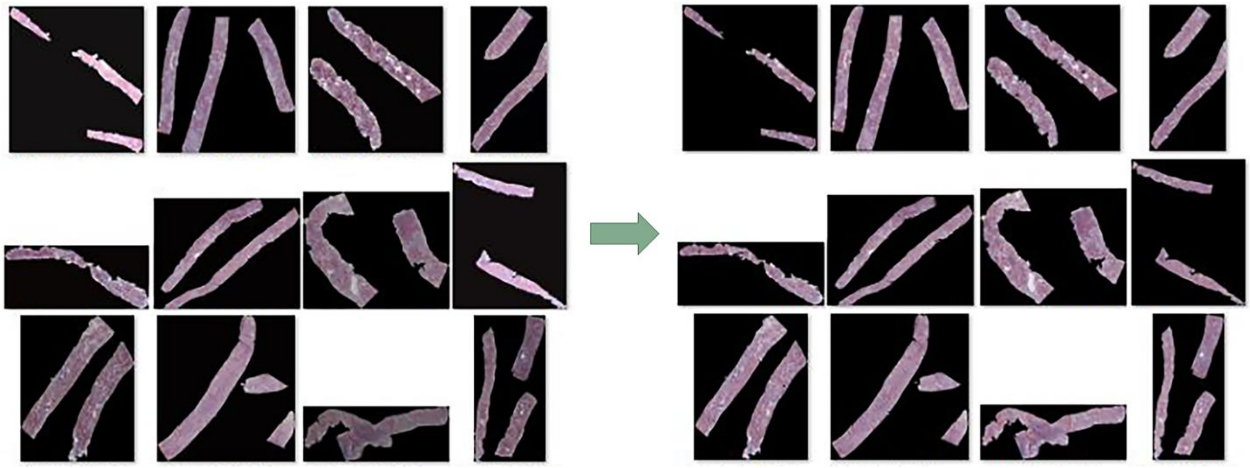

Figure S1. Stain color normalization. (A) Example images of biopsy samples. Multiple cortexes are combined in each case. To reduce the color and intensity variations present in the stained images, we computed the global mean and standard deviation of each channel in the Lab color space for the Reinhard color normalization for all data and used them as reference values to normalize our data. The figure shows Reinhard color normalization before (left) and after (right)[1].

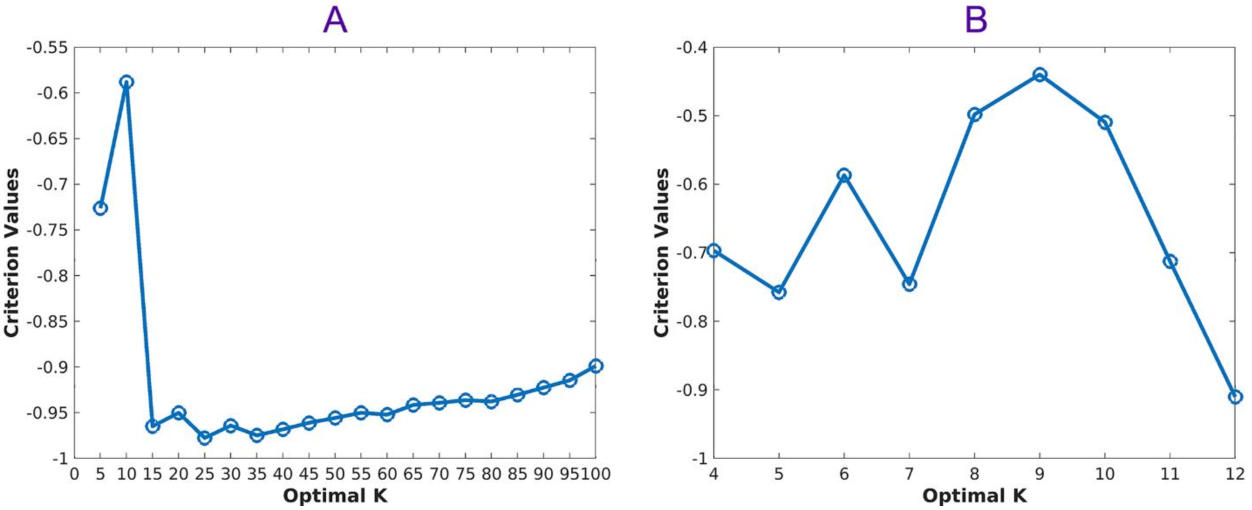

Figure S2. Determining an optimal number (K) of clusters using the Silhouette algorithm. (A) First, we run the algorithm every 5th point between 5 and 100 and then (B) run the algorithm between 4 and 12 to find the optimal K = 9 for the K means clustering[1].

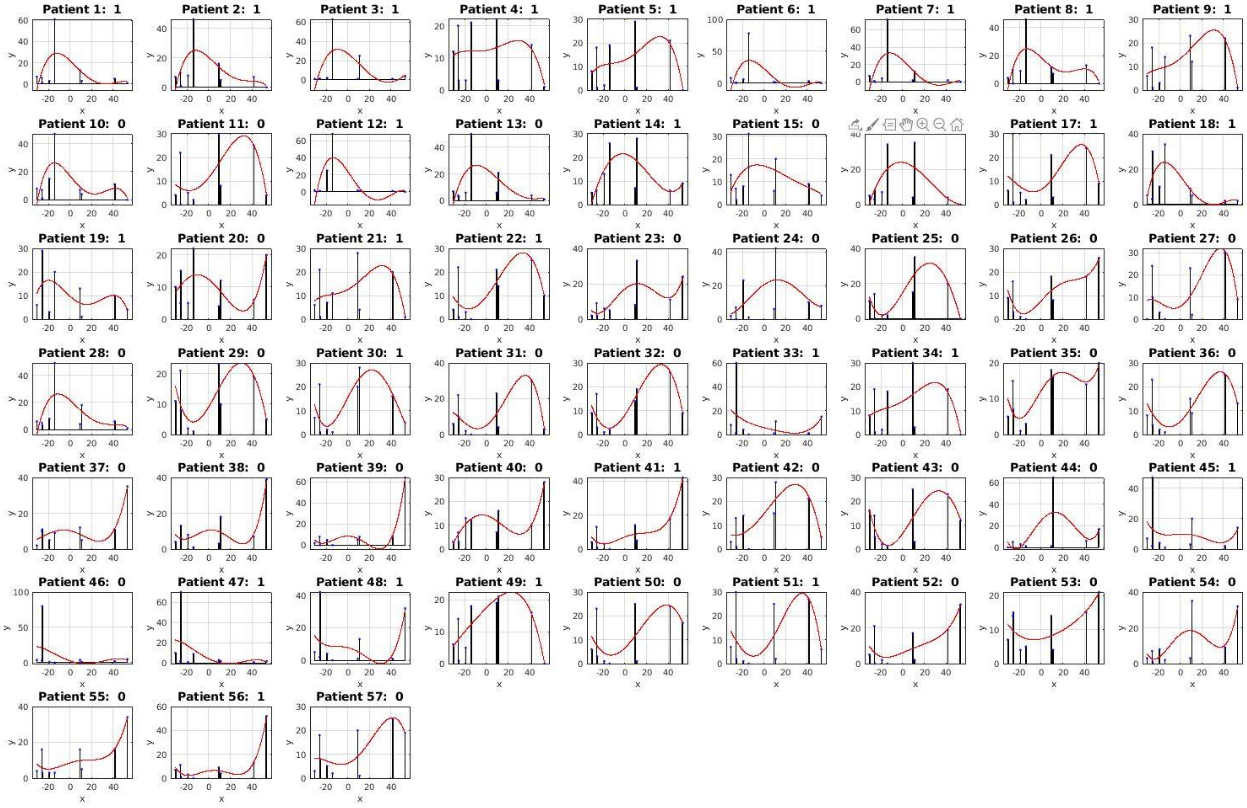

Figure S3. Frequency Histograms with the 4th polynomial fittings for all 57 cases with labels (0 for eGFR ≥ 60 and 1 for eGFR < 60). The x-axis represents the distance between cluster groups obtained from MDS and y-axis represents the normalized frequencies of the clusters[1].

References

1 Lee, J. *et al.* Unsupervised machine learning for identifying important visual features through bag-of-words using histopathology data from chronic kidney disease. *Sci Rep* **12**, 4832, doi:10.1038/s41598-022-08974-8 (2022).
